# Nutritional Management With a Casein-Based Extensively Hydrolysed Formula in Infants With Clinical Manifestations of Non-IgE-Mediated CMPA Enteropathies and Constipation

**DOI:** 10.3389/falgy.2021.676075

**Published:** 2021-06-11

**Authors:** Mikaela Sekkidou, Leilani Muhardi, Constantina Constantinou, Urszula Kudla, Yvan Vandenplas, Nicolaos Nicolaou

**Affiliations:** ^1^N Asthma and Allergy Center, Limassol, Cyprus; ^2^Friesland Campina AMEA, Singapore, Singapore; ^3^University of Nicosia Medical School, Nicosia, Cyprus; ^4^FrieslandCampina, Amersfoort, Netherlands; ^5^Kidz Health Castle University Hospital Brussels, Brussels, Belgium

**Keywords:** casein-based extensively hydrolysed infant formula, non-IgE-mediated CMPA, FPIES, FPIAP, FPE, constipation

## Abstract

**Background:** The majority of mixed-fed infants with non-IgE-mediated cow's milk protein allergy (CMPA) enteropathies are managed with an extensively hydrolysed cow's milk based infant formula (eHF). Given the high variability in peptide distribution of available eHFs, it is important to understand the suitability of a specific product in the management of distinct phenotypes.

**Objective:** To assess the symptom resolution of various phenotypes of clinical manifestations of CMPA enteropathies and constipation managed by a casein-based eHF.

**Methods:** The data of 20 full-term infants (*n* = 15 with non-IgE-mediated CMPA and *n* = 5 with constipation) attending a paediatric allergy clinic in Cyprus and managed with a casein-based eHF were retrospectively analysed.

**Results:** Based on the clinical symptoms and history, infants were classified into the following phenotypes: (a) 11/15 (73.3%) FPIAP, (b) 3/15 (20%) FPIES, and (c) 1/15 (6.7%) severe diarrhoea. Overall, 14 (93.3%) patients were successfully managed with the casein-based eHF and 1 (6.7%) required an AAF. This formula was effective in 91% of patients with FPIAP, in 100% with FPIES and with diarrhoea. Three (60%) patients with constipation responded to the eHF.

**Conclusion:** This case-series report supports the efficacy of a particular casein-based eHF for the nutritional management of non-IgE mediated CMPA enteropathies.

## Introduction

Cow's milk protein allergy (CMPA) is one of the most common food allergies in early life. Despite the lack of methodologically sound epidemiological studies estimating the exact prevalence of CMPA, current reports suggest that it affects approximately 0.5–3% of infants worldwide (1, 2).

CMPA presents with a spectrum of clinical phenotypes driven by IgE, non-IgE or both immunologic mechanisms (2). Compared to IgE-mediated, non-IgE-mediated reactions usually develop from a few hours up to several days from the time of CMP exposure and mainly involve the gastrointestinal system (3). Manifestations may vary in frequency and severity and include irritability, intense crying, colic, abdominal bloating, regurgitation, vomiting, diarrhoea, constipation, blood and/or mucus in stools, growth faltering, lethargy and hypovolemic shock (4–6). Depending on the impacted segment of the GI tract and corresponding symptoms (Appendix 1), a non-IgE-mediated allergy is classified into the following clinical entities: Food Protein induced Enteropathy (FPE), affecting the small intestine, Food Protein Induced Enterocolitis Syndrome (FPIES) affecting the entire gastrointestinal tract and Food Protein Induced Allergic Proctocolitis (FPIAP) localised to the colon (7).

Once the diagnosis of non-IgE-mediated CMPA has been confirmed, management is based on the elimination of CMP from the patient's diet, the provision of a nutritionally appropriate for age diet, and the periodic re-evaluation to assess tolerance acquisition. It is influenced by the type of patient's feeding (breastfed, mixed-fed, formula-fed) and the clinical phenotype by which CMPA presents. Breastfeeding should always be encouraged, and CMP avoidance in maternal diet may not be necessary (8, 9). Most mixed-fed/formula-fed infants would tolerate an extensively hydrolysed cow's milk formula (eHF) with proven efficacy as milk substitute and an Amino Acid Formula (AAF) could be used in severe cases (9–11) as per several international guidelines (Appendix 2).

However, not all eHF formulas are the same in term of their peptide molecular weight distribution which may affect their allergenicity and immunogenicity (12, 13). Thus, their efficacy in the management of patients with different CMPA phenotypes may differ and therefore should be assessed individually. In a recent study, Frisolac AC, an Extensively Hydrolysed Casein-based Formula produced by FrieslandCampina, did not induce proinflammatory cytokines response or T-cell proliferation in blood of allergic patients (12, 13). These characteristics may be an important factor for successful management of non-IgE-mediated enteropathies, as limiting the production of pro-inflammatory cytokines secreted by T-cells may in turn help to decrease the intestinal permeability, considered to be a potential pathomechanism (14).

### Aims of the Study

Assess the clinical course including symptom resolution among patients with non-IgE-mediated CMPA enteropathies managed by a specific casein-based eHF (Frisolac AC).

### Methodology

Data of full-term infants with clinical manifestations of enteropathies due to CMPA who were managed with a specific casein-based eHF (Frisolac AC) and had complete medical records were retrospectively analysed. An additional group of infants presenting with constipation suspected to be due to CMPA and treated with the same formula was also assessed. All subjects were born between 2015 and 2019 and attended a single paediatric allergy clinic from January 2019- June 2020 in Cyprus. The study was approved by the Cyprus National Bioethics Committee number: EEBK EΠ 2020.01.07.

#### Data Collection

The medical files of patients with CMP-related symptoms attending an allergy clinic were reviewed by the Consultant Paediatrician—Allergist (Principal Investigator/PI). The data of infants managed with the casein-based eHF were then anonymously transferred into a data collection sheet. Each patient was given a Research Identification Number (RIN) and information was extracted from each patient file and recorded in a password protected document according to the GDPR European Regulation. The matching between each patient and their respective RIN was saved in a password protected document only accessible by the PI. All data was subsequently analysed anonymously via the use of RINs.

The following information were collected: (a) age when the symptoms developed (weeks), (b) clinical presentation (type and severity of symptoms), (c) type of infant feeding (breastfeeding, mixed feeding, cow's milk formula), (d) diagnostic work-up, (e) use of eHF, (f) duration of eHF use, (g) use of AAF, (h) duration of AAF if needed, (i) duration from use of eHF to symptoms' improvement as objectively observed by diminishing or disappearance of symptoms in the out-patient clinic (j) timing and outcome of OFC (if performed), (k) age when CMPA was outgrown, (l) anthropometric measurements (focus on weight), (m) family history of allergy and (n) patient demographics.

### Statistical Analysis

Statistical Analysis was performed using IBM SPSS Statistics 24. Results are presented using descriptive statistics.

## Results

Twenty-three patients with gastrointestinal CMP-related clinical manifestations treated with the casein-based eHF and complete medical data were identified. Three infants were born premature and excluded from the analysis. Fifteen subjects with clinical manifestations of CMPA-enteropathies (FPIAP, FPIES, and severe diarrhoea) and a group of five infants presenting with constipation as main manifestation was separately examined. The full dataset and detailed history description of all subjects in this case series are presented in the Supplementary Material.

### Clinical Presentation and Diagnosis

Subjects presented to the paediatric allergy clinic having one or more symptoms/signs suggesting CMPA (Table 1). Eleven (77.3%) infants had mucus and 10 (66.7%) blood in stools, 7 (46.7%) regurgitation/ vomiting and 3 (20%) developed atopic dermatitis. Two (13.3%) subjects had a history of lethargy and 1 (6.7%) presented with diarrhoea. None (0%) of the study participants had growth faltering.

**Table 1:** Characteristics of patients with CMPA by Clinical Phenotype at time of diagnosis.

	**Clinical phenotype**				
		**FPIAP** ***n*(%)** **11 (73.3%)**	**FPIES** ***n* (%)** **3 (20%)**	**Severe** **diarrhoea *n* (%)** **1 (6.7%)**	**Total** ***N* (%)** **15 (100%)**
Patients' characteristics					
Conception method	Normal	10	2	1	13 (86.7%)
	IVF	1	1	0	2 (13.3%)
Mode of delivery	Normal	7	1	0	8 (53.3%)
	Caesarean	4	2	1	7 (46.7%)
Breastfeeding		10	3	0	13 (86.7%)
Duration of breastfeeding (mean, range weeks)		31 (1–56)	19 (8–30)	0	29 (1–56)
Formula in 1st week of life		11	1	1	13 (86.7%)
Type of feeding	Formula fed	1	0	1	2 (13.3%)
	Mixed fed	10	3	0	13 (86.7%)
Age at which symptoms developed (mean, range weeks)		7 (1–24)	6 (2–13)	1	7 (1–24)
Clinical presentation—symptoms/signs					
Regurgitations		0	0	1	1 (6.7%)
Vomiting		2	3	1	6 (40%)
Diarrhoea		0	0	1	1 (6.7%)
Blood in stools		10	0	0	10 (66.7%)
Mucus in stools		11	0	0	11 (77.3%)
Blood and mucus in stools		10	0	0	10 (66.7%)
Constipation		1	0	0	1 (6.7%)
Irritability/distressed		3	3	1	7 (46.7%)
Lethargic		0	2	0	2 (13.3%)
Eczema		2	0	1	3 (20%)
Type of infant formula used					
*Standard formula*		10	2	0	12 (80%)
*Special Infant formula*					
Partially hydrolysed formula		4	1	1	6 (40%)
Infant formula for constipation		2	1	0	3 (20%)
Anti-reflux formula		0	0	0	0 (0%)
Lactose free formula		0	0	0	0 (0%)
*Other eHF used*		3	0	0	3 (20%)
Type of other eHF used	Whey based	2	0	0	2 (13.3%)
	Casein based	0	0	0	0 (0%)
	Both whey and casein	1	0	0	1 (6.7%)

Based on the clinical manifestation and history, subjects were classified to the following three non-IgE CMPA phenotypes: (a) 11 subjects (77.33%) with FPIAP, (b) 3 (20%) with FPIES, and (c) 1 (6.7%) with severe diarrhoea due to enteropathy. The characteristics of patients by CMPA clinical phenotype are presented in Table 2. Of those with FPIAP, all cases had both blood and/or mucus in stools; with additional symptoms of: 2 (18.2%) had vomiting, 1 (9.1%) had constipation and 3 (27.3%) were distressed. All subjects with FPIES had multiple episodes of profuse vomiting 1–3 h after ingesting cow's milk formula and in 2 (66.6%) was followed by lethargy. The only infant presenting with severe diarrhoea on the 2nd day of life also developed regurgitation, vomiting, irritability and atopic dermatitis.

**Table 2:** Clinical outcomes after the nutritional management with the eHF.

	**Clinical phenotype**			
	**FPIAP** ***n* (%) 11 (73.3%)**	**FPIES** ***n* (%)** **3 (20%)**	**Severe diarrhoea *n* (%)** **1 (6.7%)**	**Total** ***N* (%)** **15 (100%)**
Subjects successfully managed with the studied casein-based eHF	10 (90.1%)	3 (100%)	1 (100%)	14 (93.3%)
Subjects switched to AAF	1	0	0	1 (6.7%)
Duration of the eHF use (mean, range months)	11.1 (7–20)	15.0	18.0	14.7 (7–20)
Duration to symptoms improvement (mean, range days)	8 (2–21)	1	4	6 (1–21)
Duration from 1st symptoms to Negative OFC (mean, range months)	11.9 (7.5–20)	19.0 (15–23)	17.0	13.3 (7.5–23)
Age at which CMA can be reintroduced (mean, range months)	14.1 (8–22)	20.8 (15–26.5)	17.0	15.3 (8–26.5)

### Age When Symptoms Developed

Subjects presented their first symptoms of the non-IgE CMPA phenotypes at a mean age of 7 weeks (range 1–24 weeks). The mean age of first presentation per phenotype were: FPIAP: 7 weeks (range 1–24 weeks) (an outlier was exclusively breastfed until the age of 24 weeks when infant formula was introduced); FPIES: 6 weeks (range 2–13 weeks); and severe diarrhoea: during the 1st week of life.

### Family History of Allergy

Twelve subjects (12/15; 80%) had at least one first degree relative with a history of allergic disease (defined as family history in this study) with maternal history being the most common (60%). Regarding the type of family history of allergy, food allergy was the most frequent (*n* = 7, 46.7%), followed by allergic rhinitis (*n* = 4, 26.7%), drug allergy (*n* = 2, 13.3%) and atopic dermatitis (*n* = 1, 6.7%). None of the subjects had a family history of allergic asthma. Five patients (33.3%) had a family history of CMPA. Twelve (80%) subjects were first born, 3 of which (20%) had no family history of allergy. Four out of the nine (44.4%) first born infants, had food allergy as the reported allergic disease.

### Birth History and Type of Infant Feeding

Conception was normal in 13/15 (86.7%) cases and 7/15 (46.7%) subjects were born via caesarean section. Eleven infants (73.3%) were mixed-fed, 2 (13.3%) were formula-fed and 2 (13.3%) were exclusively breastfed during in the 1st week of life. The average duration of breastfeeding was 29 weeks (range 1–56).

### Management With the Casein-Based Extensively Hydrolysed Infant Formula and Use of AAF

Overall, this casein-based eHF was effective in the 14/15 (93.3%) cases (Table 3). Only 1/15 (6.7%) patient with FPIAP was switched to an AAF in order to resolve his symptoms as objectively observed in the out-patient clinic. The non-responder was introduced to a partially hydrolysed formula during the 1st week of life and he developed symptoms at the end of the 1st month. His condition did not improve after 2.5 months on the eHF and was therefore switched to AAF. Interestingly, three of the patients who responded to the casein eHF had previously been fed other eHFs, of which one was a casein eHF as well. All three with FPIES and the one with severe diarrhoea responded well to this casein-based eHF.

**Table 3:** Characteristics of patients with constipation due to suspected CMPA.

**Patients' characteristics**		***N* (%) 5 (100%)**
Conception method	Normal	3 (60%)
	IVF	2 (40%)
Mode of delivery	Normal	0
	Caesarean	5 (100%)
Breastfeeding		5 (100%)
Duration of breastfeeding (mean, range weeks)		16 (3-30)
Formula in 1st week of life		5 (100%)
Type of feeding	Formula fed	0
	Mixed fed	5 (100%)
Age at which symptoms developed (mean, range weeks)		2 (1-3)
Clinical presentation—symptoms/signs		
Regurgitations		2 (40%)
Vomiting		0
Diarrhoea		1 (20%)
Blood in stools		0
Mucus in stools		0
Blood and mucus in stools		0
Constipation		5 (100%)
Irritability/distressed		5 (100%)
Lethargic		0
Eczema		0
Type of infant formula used		
*Standard formula*		4 (80%)
*Special infant formula*		
Partially hydrolysed formula		3 (60%)
Infant formula for constipation		2 (40%)
Anti-reflux formula		1 (20%)
Lactose free formula		1 (20%)
*Other eHF used*		0
Type of other eHF used	Whey based	0
	Casein based	0
	Both whey and casein	0
Subjects successfully managed with the studied casein-based eHF		3 (60%)
Subjects switched to AAF		2 (40%)
Duration of the eHF use (mean, range months)		8.3 (8–8.5)
Duration to Symptoms Improvement (mean, range days)		8 (7–10)
Duration from 1st symptoms to Negative OFC (mean, range months)		13.0 (11–16.5)
Age at which CMA can be reintroduced (mean, range months)		13.4 (12–17)

### Time Frame for Clinical Improvement

The average time for clinical improvement as defined for all the clinical phenotypes was 6 days (range 1–21) after switching to the casein-based eHF. In infants with clinical manifestations of FPIAP, improvement was observed at a mean of 8 days (2–21), all subjects with FPIES from the 1st and the infant with severe diarrhoea after 4 days of treatment.

### Duration and Resolution of CMPA

The average time from clinical presentation to re-introduction of standard cow's milk formula/fresh milk into diet without problems in all cases was 13.3 (range 2.5–23) months [FPIAP 11.9 (7.5–20) months, FPIES 19 (15–23) months, severe diarrhoea 17 months]. The re-introduction of standard cow's milk formula was first conducted in the out-patient clinic under supervision of the paediatric allergist. One subject with FPIES had not outgrown CMP by the age of 17 months. CMP was successfully reintroduced in seven infants (46.7%) between 12 and 14 months of age, in 4 (26.7%) between 15 and17 months and in 2 (13.3%) between 22 and 27 months. The one patient who was classified as FPIAP and required an AAF, outgrew CMA at the age of 14 months.

### Characteristics of Subjects Presenting With Constipation and Managed With the Casein-Based eHF

All 5 (100%) subjects with constipation developed during the first 3 weeks of life were born via caesarean section and mixed-fed from the 1st week of life (Table 2). Three (60%) infants had a family history of CMPA. Four (80%) infants who were referred from other paediatric clinics at which were initially managed with other special infant formulas (partially hydrolysed, specific for constipation, anti-reflux, lactose-free). After switching to the study eHF, three (60%) had successfully improved in stool consistency and frequency; the mean duration for clinical improvement was 8 days (range 7–10). The mean duration form initial presentation to resolution was 13 months (11–16.5). In two subjects (40%) symptoms resolved only after switching to an AAF.

## Discussion

In this retrospective study the clinical course and resolution of symptoms of a specific casein-based extensively hydrolysed infant formula in subjects presenting with clinical manifestations of non-IgE-mediated CMPA enteropathies in a paediatric allergy clinic was analysed. Overall, this formula was effective in the management of 93.3% of the cases. Ten out of 11 (90.1%) infants with FPIAP, all three with FPIES (100%) and the one with severe diarrhoea were successfully managed with this casein-based eHF. With regards to FPIAP, only 1 (9.1%) patient required an AAF to resolve his symptoms, whereas in a recent USA study 22.6% were managed with an AAF (15). Given that only one subject in this case series needed an AAF, no further specific characteristics for those who required an AAF could be identified.

As such, the rate of effectiveness of this product in managing FPIAP, FPIES and severe diarrhoea due to CMPA met the criteria of the International Scientific Organisations Guidelines (including the American Academy of Paediatrics and the European Academy of Allergy and Clinical Immunology) for the declaration of an infant formula as hypoallergenic to be effective in 90% of infants with CMPA (16–18). This is in line with a number of current guidelines on the management of non-IgE-mediated CMPA (Appendix 2), suggesting the use of an eHF as the 1st choice treatment in FPIAP and diarrhoea and the use of AAF in FPIES and other severe non-IgE-mediated CMPA cases. However, AAF has been reported to lack the capability of building up tolerance and imposes high economic burden on families and healthcare systems (5, 19, 20). In addition, as the average duration of formula use in this case studies was longer than 6 months, prolonged use of AAF could increase the risk of hypophosphatemia and rickets as reported earlier (20–24). Therefore, clinicians should limit its use for extremely severe cases and when eHF is not tolerated, which is supported by this study findings (9, 25, 26).

Not all hydrolysed infant formulas are the same (12, 13). Their hypoallergenicity depends on the specific condition of the hydrolysis process (i.e., duration, temperature, type of enzyme used etc.), the protein used (i.e., whey or casein) and the overall production process of final formula. These variations result in different residual epitopes and molecular weight of the peptides. Hence, the allergenicity of individual eHFs may be significantly different reflecting different level of tolerance by patients with CMPA (13). Other components in the infant formula (i.e., probiotics, prebiotics, fatty acids, oligosaccharides etc) may also play a role in their effectiveness and support building of oral tolerance. (13, 17, 27–30). In the present study, 3 of 15 (20%) infants had previously tried other eHFs with no success. All three cases had tried a whey-based eHF and one also tried another casein-based eHF. Only the studied casein-based eHF led to the resolution of their symptoms which may be directly related to lower allergenicity of this formula compared to those used before (13).

The time period from elimination of the CMP in the patient's diet to improvement and resolution of symptoms in this report (mean 6 days; range 1–21 days) was shorter than those reported in earlier studies, which varied from 1 day to 4 weeks depending on the clinical presentation and severity of CMPA (10, 31). This fast improvement despite CMPA severity may be related to the fact that all cases had access to immediate specialist care and appropriate management.

The mean age at which infants outgrew CMPA was 15.3 months (range 8–26.5) which is commonly observed in patients with non-IgE-mediated CMPA (3, 32, 33). Seven (46.7%) outgrew CMPA between 12–14 months of age and 4 (26.7%) between 15–17 months. Particularly in FPIAP the mean age was 11.9 months in agreement with recent reports (33). The patient who outgrew CMPA by the age of 8 months was diagnosed with mild FPIAP at the age of 2 weeks suggesting that early diagnosis and appropriate management may result in earlier tolerance acquisition.

Another infant who outgrew CMPA at the age of 22 months was the one in which parents tried to reintroduce cow's milk into her diet twice without the guidance of a specialist. This may have resulted in recurrent and prolonged inflammation of the GI tract and further delay in the development of tolerance. While the one who outgrew CMPA at the age of 26.5 months could have probably become tolerant earlier but, his mother was reluctant to proceed to a supervised reintroduction with a standard formula. In an effort to reduce such phenomena, it is important that healthcare providers involved in the management of patients with CMPA educate and support parents and caregivers appropriately (9, 34). Unevidenced advice and recommendations (i.e., by “Dr. Google” or other parents with similar experiences) should be avoided by the parents (9).

In this paediatric allergy clinic, infants with CMPA are usually reviewed between the age of 9 and 12 months and IgE sensitisation to CMP is assessed by Skin Prick Test or specific IgE in serum (especially in those with immediate-type symptoms, FPIES and Atopic Dermatitis) in accordance to international guidelines (24, 35). If the tests results are negative, then gradual reintroduction of cow's milk in the infant's diet is performed according to the Milk Ladder protocol (9, 10, 24), except in the case of FPIES where a controlled OFC is preformed 12 months after initial diagnosis. CMPs are introduced initially in baked form. Infant formula and fresh milk which consist intact protein is reserved as the last step. In general, a duration of two months is usually needed to reach intact cow's milk consumption (36). However, the willingness of the family to proceed with CMP reintroduction and OFC procedures varies which may alter the timing of the challenges. This also explains why age at starting Milk Ladder and age at outgrown CMPA may differ.

The overall mean duration of CMPA in this study from the time of diagnosis to the age of complete CMPA resolution (infant formula with intact protein or fresh cow's milk) was 13.3 (7.5–23) months with those with FPIAP phenotype showing the shortest duration (11.9 months). The mean duration for those infants with FPIES who outgrew CMPA was 20 (15–26.5) months. Regarding the patient with FPIES who has not outgrown CMPA at the age of 17 months, his mother tried to reintroduce a standard infant formula at the early age of 4 months while infant was on an elimination diet. These observations could assist clinicians to decide when to proceed to an OFC or start the Milk Ladder procedure.

In the three cases of FPIES, two subjects developed symptoms of profuse vomiting followed by lethargy after the first feedings of infant formula (ages 2 and 4 weeks) which could be considered as severe. The other subject was mixed-fed with a standard infant formula for 3 months (age 13 weeks) until he developed the first symptoms. All three infants were mixed-fed and none of their mothers had to follow a dairy elimination diet for improvement of symptoms. Several guidelines (10, 11, 24, 37, 38), suggest an AAF as the 1st choice for the management of FPIES. However, in a recent Spanish study (36), only 20% of infants with FPIES needed to switch to an AAF for their symptoms to resolve. This reflects our findings where none of infants with FPIES required an AAF. Despite the small number of cases in our series, we cannot ignore the fact that all three infants were successfully managed with the casein based eHF and continuation of breastfeeding without exclusion of dairy products in maternal diet. This may suggest that all mixed-fed infants with FPIES in addition to the encouragement of breastfeeding should primarily be treated with an eHF and that AAF could be suggested if eHF is not tolerated (9, 26) and a randomised-double blind control trial of eHF vs. AAF may be needed to confirm this hypothesis in the future.

One of the most interesting findings in this study is the association between the family history of allergy and the risk of developing CMPA. Family history of any allergy, food allergy and CMPA was present in 80, 46.7, and 33.3% of subjects, respectively. Maternal history of allergy appeared more prevalent than paternal history of allergy (60 vs. 13.3%). In a recent Chinese study (39), the strong correlation of family history of food allergy and especially maternal food allergy was also observed. Despite the small number of participants, we observed a relation between the presence of CMPA history in the family and the development of CMPA as 1 in 3 patients in this study had a family member with CMPA. Studies conducted in Italy and USA reported that an atopic family history was present in 65–79% of patients with FPIES (40, 41) and in around 36% of patients with FPIAP (42). Three had chronic FPIES turning into acute FPIES. Cow milk was the most common triggering food (50%), followed by fish (21.4%). Therefore, the presence of CMPA in family history may be an important risk factor for the development of CMPA. Emphasis is usually given on the presence of atopic dermatitis in the family as a risk factor for CMPA (9, 43). However, this was not the case in our study population where family history of atopic dermatitis was present in only one subject.

Recent publications suggest that supplementation with a cow's milk formula in the 1st week of life followed by discontinuation of this formula for extended period of time, may be another risk factor for CMPA (44, 45). In our case series, 80% of infants were fed an intact cow's milk infant formula during the 1st week of life further supporting this observation. However, it would be interesting to see whether the use of a partially hydrolysed formula in the 1st week of life in mixed fed infants could reduce the risk of CMPA development in future studies.

Breastfeeding should always be encouraged in patients with CMPA. Seven out of 11 infants with FPIAP were exclusively or partially breastfed at the time of diagnosis and all improved after dairy exclusion in maternal diet and supplementation with the casein-based eHF. One subject with FPIAP was exclusively breastfed for the first 2 weeks of life when he switched to mixed feeding with a pHF and developed blood in stools. After breastfeeding was discontinued, his clinical condition worsened and improved only when switching to an AAF, suggesting a possible protective effect of breast milk.

Regarding the five infants with constipation that were managed by CMP-elimination diet using the casein-based eHF, 3 (60%) were successfully managed and 2 (40%) had to switch to an AAF in order to resolve their symptoms. It is interesting that in responders symptoms resolved at a mean of 8 days (range 7–10) after casein-based eHF administration. Although the percentage of non-responders may be considered high for these cases, we could not suggest AAF as the 1st choice treatment for patients with constipation as it is not usually severe or life-threatening. On the other hand, the majority of these infants were successfully treated with the casein-based eHF. However, duration and severity of CMP-related constipation should be considered as well as its impact on the patient's quality of life. It is worth noticing that all 5 infants with constipation were born via caesarean section. One could speculate that the development of a different intestinal microbiome in these infants compared to those born via the vaginal route (46) may confer susceptibility to an increased risk of CMP-related constipation as observed in this group of infants.

Published data for constipation related to CMPA in infancy are limited and most refer to older children (47, 48). The Rome IV criteria are mainly focused on dyschezia and functional constipation for infants and toddlers and toilet and non-toilet-trained toddlers, not including CMP as the cause of the defecation difficulty (49). A recent French study (50) including infants up to 12 months of life, showed that constipation diagnosis made clinically by physicians was doubled compared to diagnosis made by applying the Rome IV criteria, indicating the gap in constipation diagnosis in early infancy. It is reported that for the cases where CMP-related constipation is suspected, an elimination diet followed by food provocation should be performed in order to confirm diagnosis. With the exception of DRACMA (35), guidelines on CMPA do not address management of constipation due to CMPA and the possible use of eHF in this condition.

### Limitations of the Study

This study is a small sample size, single centre, retrospective, observational study exploring the effectiveness of only 1 type of eHF; while the majority of infants with CMPA in Cyprus are usually managed with another type of eHF which is provided for free by the public health care system. Thus, we cannot compare the effectiveness of this casein-based eHFs with other eHFs in terms of symptoms resolution in the same set-up. To date, there are no studies comparing two different types/brands of eHF and it would be very interesting if this kind of study could be conducted in the future (27). In addition, the number of studies using eHF for nutritional management of non-IgE-mediated gastroenteropathies are quite limited, probably due to the low prevalence of the disease.

Another limitation is that medical diagnosis for CMPA was made according to the detailed medical history and presence of clinical manifestation. It was not confirmed with OFC, except for two cases. Although DBPCFC is considered the Gold Standard for CMPA diagnosis, in daily clinical practise, its performance may be difficult, time-consuming and expensive and it is rarely applied (14, 51). On the other hand, a thorough medical history and clinical examination taken by a practitioner experienced in food allergy, is a commonly accepted tool for differentiation and diagnosis of CMPA (10).

## Conclusion

The casein-based eHF administered in this study was effective in more than 90% of patients with clinical manifestations of FPIAP, 100% with FPIES and 100% with severe diarrhoea. Although casein-based eHF has not been designed for the management of constipation, in this small number of patients, it was helpful in the majority of subjects with CMP-related constipation. Therefore, this casein-based eHF could be suggested as a 1st choice milk substitute for the management of non-IgE-CMPA enteropathies and when constipation is considered as CMPA-related. Different allergenicity profile of this casein-based eHF may contribute to treatment effectiveness for FPIES.

Applying the most appropriate individualised nutritional management plan for every patient with gastrointestinal manifestations of non-IgE-mediated CMPA remains challenging. Further studies are required to establish the efficacy, safety of long-term use, and explore the ability to induce oral tolerance of different hypoallergenic formulas in confirmed enteropathies phenotypes.

## Data Availability Statement

The original contributions generated for this study are included in the article/Supplementary Material, further inquiries can be directed to the corresponding author/s.

## Ethics Statement

The studies involving human participants were reviewed and approved by Cyprus National Bioethics Committee number: EEBK EΠ 2020.01.07.

## Author Contributions

MS analysed the data and drafted the manuscript. LM and UK were involved in the design and reviewed the manuscript. CC contributed to the design and statistical analysis. YV reviewed the manuscript. NN extracted the data, reviewed clinically all the cases and supervised the writing of the manuscript. All authors accepted the final version of the manuscript.

## Conflict of Interest

MS is a research assistant and NN the Principal Investigator of the Allergy Reduction Trial (A.R.T.) in Cyprus: The cow's milk protein allergy risk reducing effect of Frisolac Gold preventive HA: Multicenter, Placebo-Controlled trial, sponsored by Friesland Campina and conducted at the N Asthma and Allergy Centre. LM and UK are employees of Friesland Campina at the time of drafting and submitting the manuscript. The remaining authors declare that the research was conducted in the absence of any commercial or financial relationships that could be construed as a potential conflict of interest.

## Abbreviations

AAF: Amino Acid Formula
CMP: Cow's Milk Proteins
CMPA: Cow's Milk Protein Allergy
eHF: Extensively Hydrolysed infant Formula
FPE: Food Protein Enteropathy
FPIAP: Food Protein Induced Allergic Proctocolitis
FPIES: Food Protein Induced Enterocolitis Syndrome
OFC: Oral Food Challenge.
