# Supplementary material for: Nutritional Management With a Casein-Based Extensively Hydrolysed Formula in Infants With Clinical Manifestations of Non-IgE-Mediated CMPA Enteropathies and Constipation
Source: Front Allergy. 2021 Jun 11;2:676075. doi: 10.3389/falgy.2021.676075 (PMC8974831; doi:10.3389/falgy.2021.676075)
Supplement: Supplementary file 1 [file Data_Sheet_1.pdf]

Appendix 1 *- Diagnostic criteria for non-IgE mediated CMPA Enteropathies* [14,51]

**FPIAP**	**FPIES**	**Severe Diarrhoea/ FPE**
Small amount of rectal bleeding in an otherwise healthy infant	<2 years of age at first presentation	< 9 months of age at the initial diagnosis
Disappearance of the symptoms after all antigens are removed from the diet.	Exposure to inciting food elicits repetitive and projectile vomiting, lethargy within 2-4 hours	Repeated exposure to causative food elicits GI symptoms without alternative cause, predominantly vomiting and failure to thrive
Exclusion of other cause of rectal bleeding	Usually resolve within 6-h	Confirmation of the diagnosis by small bowel biopsy
	Diarrhoea may present, much less frequently and later (5-10 h)	Removal of causative food results in resolution of symptoms within several weeks, although complete healing of villous injury may take several months
	Avoidance of offending protein from diet results in resolution of symptoms	
	Re-exposure or Oral Food Challenge (OFC) elicits typical symptoms within 2-4 hour; two typical episodes are needed to establish the definitive diagnosis without the need to perform OFC	

Appendix 2 *- Current recommendations on nutritional management of non-IgE CMPA enteropathies in formula-fed infants*

	**Clinical Presentation**							
	**FPIAP**		**FPIES**		**Diarrhoea / Enteropathies**		**GI symptoms related to CMPA**	
**Guideline**	1st choice	2nd choice	1st choice	2nd choice	1st choice	2nd choice	1st choice	2nd choice
**ASCIA**[52]	eHF or rice formula or soy formula if >6mo	AAF	eHF or rice formula or soy formula if >6mo	AAF	eHF or rice formula or soy formula if >6mo	AAF	eHF/soy	AAF/eHF
**Spanish Association of Paediatrics**[10]	eHF	AAF	AAF		eHF (AAF in severe cases)	AAF	eHF	AAF
**DRACMA**[11]	eHF	AAF	AAF	eHF (if AAF refusal)	eHF	AAF	eHF	AAF
**BSACI**[24]	eHF		AAF		eHF (AAF if severe)	AAF		
**EAACI**[53]	eHF	AAF	AAF	-	AAF	-	-	-
**Middle East**[54]	eHF	AAF	eHF	AAF	eHF	AAF	eHF	AAF
**ESPGHAN**[55]	eHF	AAF	eHF	AAF	eHF or AAF in severe enterop athy	AAF	eHF	AAF
**MSAI & PPM**[56]	eHF	AAF	eHF	AAF	eHF	AAF	eHF	AAF
**Italian Guidelines**[38]	eHF	AAF	AAF	eHF	eHF	AAF	eHF	AAF
**American Academy of Allergy, Asthma & Immunology (AAAAI)**			eHF	AAF				
**American College of Allergy, Asthma & Immunology (ACAAI)**<br><br>*This page was reviewed and updated as of 3/21/2019*			Casein-based hydroly sate					

Appendix 2. Current international recommendations of associations specialized in gastrointestinal disorders and/or allergies regarding the 1st and 2nd choice of infant formula used in the treatment of CMPA gastrointestinal food allergies. In all, an eHF is the 1st choice of treatment for FPIAP, diarrhoea, constipation and GI symptoms related to CMPA. For patients with FPIES, DRACMA, ESPGHAN, BSACI, EAACI, Spanish and Italian suggest an AAF as the first choice of treatment.

Reference for Appendix 2:

10. Espín Jaime, B.; Díaz Martín, J.J.; Blesa Baviera, L.C.; Claver Monzón, Á.; Hernández Hernández, A.; García Burriel, J.I.; Mérida, M.J.G.; Pinto Fernández, C.; Coronel Rodríguez, C.; Román Riechmann, E.; et al. Non-IgE-mediated cow's milk allergy: Consensus document of the Spanish Society of Paediatric Gastroenterology, Hepatology, and Nutrition (SEGHNP), the Spanish Association of Paediatric Primary Care (AEPAP), the Spanish Society of Extra-hospital Paediatric. An. Pediatr. 2019, 90, 193.e1-193.e11, doi:10.1016/j.anpedi.2018.11.007.

11. Fiocchi, A.; Dahda, L.; Dupont, C.; Campoy, C.; Fierro, V.; Nieto, A. Cow's milk allergy: towards an update of DRACMA guidelines. World Allergy Organ. J. 2016, 9, 1–11, doi:10.1186/s40413-016-0125-0.

24. Luyt, D.; Ball, H.; Makwana, N.; Green, M.R.; Bravin, K.; Nasser, S.M.; Clark, A.T. BSACI guideline for the diagnosis and management of cow's milk allergy. Clin. Exp. Allergy 2014, 44, 642–672, doi:10.1111/cea.12302.

38. Díaz, J.J.; Espín, B.; Segarra, O.; Domínguez-Ortega, G.; Blasco-Alonso, J.; Cano, B.; Rayo, A.; Moreno, A. Food Protein-induced Enterocolitis Syndrome: Data From a Multicenter Retrospective Study in Spain. J. Pediatr. Gastroenterol. Nutr. 2019, 68, 232–236, doi:10.1097/MPG.0000000000002169.

52. Australiasian society of clinical immunology and allergy (ASCIA) Guide for Milk Substitues in Cow's Milk Allergy Available online: https://www.allergy.org.au/images/stories/pospapers/ASCIA_HP_Guide_CMA_Milk_Substitutes_2020.pdf.

53. EAACI Food Allergy and Anaphylaxis Guidelines: Supplementary materials. Translating knowledge into clinical practice. Eur. Acad. Allergy Clin. Immunol. 2014, 188.

54. Vandenplas, Y.; Abuabat, A.; Al-Hammadi, S.; Aly, G.S.; Miqdady, M.S.; Shaaban, S.Y.; Torbey, P.H. Middle east consensus statement on the prevention, diagnosis, and management of cow's milk protein allergy. Pediatr. Gastroenterol. Hepatol. Nutr. 2014, 17, 61–73, doi:10.5223/pghn.2014.17.2.61.

55. Koletzko, S.; Niggemann, B.; Arato, A.; Dias, J.A.; Heuschkel, R.; Husby, S.; Mearin, M.L.; Papadopoulou, A.; Ruemmele, F.M.; Staiano, A.; et al. Diagnostic approach and management of cow's-milk protein allergy in infants and children: Espghan gi committee practical guidelines. J. Pediatr. Gastroenterol. Nutr. 2012, 55, 221–229, doi:10.1097/MPG.0b013e31825c9482.

56. Khoh, A.; Mun, K.; Hamzah, A.; Latiff, A.; Chai, D.; Fan, P.; Selayang, P.H.; Caves, B.; Darul, S.; Chong, E.D.; et al. Committee members Dr Cheong Hon Kin. 2012, 2012.

Appendix 3 *- Full Dataset and Detailed History in subjects with CMPA*

CSCy ID	Gender	Type of feeding	Conception	Mode of Delivery	Family History of Allergy	Number of family members with history of allergy	Number of family members with food allergy	Previous Formula	Cardinal Symptom	Clinical Presentation	Age at symptoms onset (weeks)	Duration of symptoms improvement after introducing Frisolac AC (days)	Duration of Frisolac AC use (months)	Use of AAF	Age at starting Milk Ladder (months)	Age outgrown CMPA (months)	Duration from 1st symptoms to Negative OFC (months)
CSCy01	Female	Mixed fed	Normal	Normal	Mother with drug allergy to Penicillin	1	0	Standard formula; eHF (whey based and casein based)	Blood and mucus in stools	Blood in stools; Mucus in stools; Vomiting; Eczema	6	2	9.5	NO	11	14	11
The infant was mixed fed for the 1st week of life with a Standard Formula and then she was exclusively breastfed until the age of 5 months. During the first weeks of life the infant had eczema and she was vomiting with every feeding. Ranitidine administration did not improve her vomiting. At the age of 6 weeks she had blood and mucus in stools. Her mother followed an elimination diet excluding dairy products and symptoms improved. At the age of 4.5 months the infant had again blood and mucus in stools and her mother tried to introduce an extensively hydrolysed whey-based formula as complementary feeding but symptoms did not improve. Therefore, the infant formula was switched to Frisolac AC and symptoms improved within 2 days. She outgrew CMPA by the age of 12 months but her mother continued feeding her with Frisolac AC until the age of 14 months.																	
CSCy02	Female	Mixed fed	Normal	Caesarean	Mother with drug allergy to Antibiotic	1	0	Standard formula	Blood and mucus in stools	Blood in stools; Mucus in stools; Vomiting; Eczema	1	7	14	NO	13	14	13
The infant was mixed fed from birth with a Standard Formula. At the age of 10 days she developed blood and mucus in stools. Therefore, the formula was switched to Frisolac AC and mother excluded dairy products from her diet. At the age of 12 months the mother tried to introduce a Partially Hydrolysed Formula but the patient had vomiting with every feeding. Therefore, she continued on Frisolac AC until assessment in the allergy clinic. She outgrew CMPA at the age of 14 months.																	
CSCy03	Female	Mixed fed	Normal	Normal	Mother with Allergic Rhinitis	1	0	Standard Formula; Partially Hydrolysed formula	Blood and mucus in stools	Blood in stools; Mucus in stools	11	14	2	NO	12	14	10
The infant was exclusively breastfed up to the age of 2 months when a Standard Formula was added as supplement. At the age of 2.5 months, she developed blood and mucus in stools. At the age of 3 months the infant formula was switched to a Partially Hydrolysed Formula. Symptoms improved however, occasionally she had mucus and flakes of blood in her stools. At the age of 5.5 months the patient developed an episode of gross blood in stools. She was admitted to hospital and paediatric surgery assessment did not reveal any significant findings. The formula was switched to Frisolac AC and the breastfeeding mother avoided dairy products into her diet. Symptoms resolved completely in the following two weeks. She discontinued Frisolac AC 2 months later and continued breastfeeding up to the age of 14 months when she outgrew CMPA.																	

CSCy04	Male	Mixed fed	Normal	Normal	Mother with Food Allergy to sesame	1	1	Partially Hydrolysed formula	Blood and mucus in stools	Blood in stools; Mucus in stools; Irritability/ Distress; Sleep disturbance, Bloating	4	14	2.5	YES	13	14	13
For the first 2 weeks of life he was exclusively breastfed and then continued mixed feeding with a Partially Hydrolysed Formula. At the age of 4 weeks he had blood and mucus in stools, irritability/distress, sleep disturbance and bloating. He was switched to Frisolac AC and improved over the following 2 weeks. However, after discontinuing breastfeeding at the age of 4 months, symptoms reappeared with gross blood in stools. Therefore, formula was switched to AAF up to the age of 14 months when he outgrew CMPA.																	
CSCy05	Male	Mixed fed	Normal	Normal	None	0	0	Standard formula; Partially Hydrolysed Formula; eHF (whey based)	Blood and mucus in stools	Blood in stools; Mucus in stools; Irritability/ Distress	5	7	10	NO	12.5	12.5	11
The infant was mixed fed for the first 24 hours of life and was EBF for 3 weeks. Then, he was mixed fed with a standard formula. At the age of 4 weeks he developed significant distress and irritability that improved with a Partially Hydrolysed Formula. However about 10 days later he developed blood and mucus in stools (3 days before appearance of symptoms he had the Rotavirus oral vaccine) and he was admitted to the hospital. He was given an extensively hydrolysed whey-based infant formula and his symptoms improved. However, after about 10 days his condition deteriorated again with blood in stools. He was switched to Frisolac AC and his symptoms improved remarkably within a week but he continued to have some mucus in stools for a few more weeks. He continued on Frisolac AC up to the age of 12.5 months when he outgrew CMPA.																	
CSCy06	Male	Mixed fed	Normal	Caesarean	Mother with Atopic Dermatitis	1	0	Standard formula	Blood and mucus in stools	Blood in stools; Mucus in stools	24	N/A	8	NO	14.5	15	9.5
The infant was mixed fed for the first 48 hours of life and then was EBF for 5.5 months. Mother tried to introduce standard infant formula in addition to breast milk and the infant developed blood and mucus in stools. She continued breastfeeding (mum did not exclude dairy from her diet) and supplemented with Frisolac AC (1 meal per day). Symptoms resolved after about a few days. However, a month later he developed significant blood and mucus in stools. Mum was advised to exclude cow's milk protein from her diet and she continued mixed feeding (breastfeeding + Frisolac AC). Symptoms resolved in a week. Breastfeeding was discontinued at age 8 months and he continued with Frisolac AC up to 15 months of age.																	
CSCy07	Male	Mixed fed	Normal	Normal	Mother with hypersensitivity to cow's milk and Brother with CMPA	2	2	Standard formula; eHF (whey based)	Blood and mucus in stools	Blood in stools; Mucus in stools	9	10	11	NO	15	15.5	13
Infant was on EBF for 3 months but at the 2nd day of life he had 2 supplementary feedings of standard formula. At the age of 2 months the infant developed loose green stools with mucus and blood stain and mother excluded dairy products from her diet. He improved within the following 2 weeks. At the age of 3 months he was supplementary fed with an extensively hydrolysed whey-based infant formula and at the 8th day of feeding he developed blood and mucus in stools. He continued with EBF up to the age of 4 months when his mother introduced Frisolac AC without any adverse event. Moreover, mucus in stools improved significantly within the first 2 weeks of Frisolac AC administration. Breastfeeding discontinued at the age of 12 months. He was on Frisolac AC up to the age of 15.5 months when he outgrew CMPA.																	

CSCy08	Male	Formula fed	IVF	Caesarean	None	0	0	Standard formula; Other type of infant special formula for constipation	Blood and mucus in stools	Blood in stools; Mucus in stools; Constipation	1	21	13.5	NO	14	14	14
This infant was exclusively formula fed with a standard formula up to the age of 1 week when he developed constipation. A special formula for constipation was added and his motions improved however, containing some mucus. At the age of 2 weeks he developed blood in stools and occult blood test was positive. He was switched to Frisolac AC and after 3 weeks no further blood and mucus were observed in his stools. He was on Frisolac AC until the age of 14 months when he outgrew CMPA.																	
CSCy09	Female	Mixed fed	Normal	Normal	Brother with CMPA	1	1	Standard formula	Blood and mucus in stools	Blood in stools; Mucus in stools	6	4	20	NO	22	22	20
The infant was EBF up to the age of 1.5 month when a standard infant formula was added into her diet. In the following days she developed mucus and blood in stools. Mum was advised to avoid dairy products into her diet and Frisolac AC was given as supplementary formula. Symptoms improved within 4 days. At the age of 6 months mum discontinued breastfeeding and tried to reintroduce standard cow's milk formula into the infant's diet. The infant developed blood in stools and therefore, she continued with Frisolac AC. AT the age of 15 months mum tried to introduce cow's milk in the form of infant cereals cream with milk and after a week the patient developed blood and mucus in stools that resolved with exclusion of cow's milk in any form from her diet. At the time she was tolerating cereals. She was on Frisolac AC up to the age of 22 months when she outgrew CMPA.																	
CSCy10	Male	Mixed fed	Normal	Caesarean	Mother with food allergy to peach, Allergic rhinitis and allergy to cats	1	1	Standard formula	Blood and mucus in stools	Blood in stools; Mucus in stools	9	2	7	NO	11	12	9
The infant was EBF and he had 2-3 complementary feedings with standard formula up to the age of 3 months. At the age of 2 months he developed blood in his stools. Mother excluded dairy products from her diet but the infant had still blood in stools. At 3 months of age mother tried to introduce an extensively hydrolysed whey-based infant formula but the infant had again blood in his stools on the same day. At the age of 4 months mother consumed accidentally cow's milk protein and the infant had again blood in stools and some mucus. Mother continued excluding dairy products from her diet and the infant was complementary fed with Frisolac AC. Symptoms resolved within 2 weeks of Frisolac AC administration. At the time, he is on Milk Ladder and tolerates baked milk in cake and foods, butter and melted cheese.																	
CSCy11	Male	Mixed fed	Normal	Normal	None	0	0	Standard infant formula; Other type of special infant formula with hydrolysed protein	Mucus in stools	Mucus in stools; Irritability/ Distress	2	7	7	NO	8	8	7.5
The infant was breastfed for 2 days and then he was exclusively formula fed with a standard formula. At the age of 2 weeks he developed significant irritability and discomfort with some mucus in stools. Formula was switched to another type of infant formula for colics and regurgitations containing partially hydrolysed whey proteins. Symptoms improved but a week later relapsed again with excessive mucus in stools. Therefore, formula was switched																	

to Frisolac AC and symptoms resolved within 1 week. The infant had 1-2 more times mucus in stools. He continued on Frisolac AC until the age of 8 months when cow's milk in form of infant cereal cream was reintroduced in his diet.																	
CSCy12	Male	Mixed fed	IVF	Caesarean	Mother with Allergic Rhinitis	1	0	Standard formula	Vomiting (FPIES)	Vomiting; Irritability/ Distress; Lethargy	2	1	15	NO		15	15
The infant was mixed fed with Frisolac AC for 2 weeks as he was taking part in a research study. At the 2nd week of life, he had to switch to Standard Formula but he refused to drink it, consuming only 30mL. About 1.5 hour later he was distressed and vomited the whole quantity. Then he fell asleep and when he woke up, he had breastfeeding without any problem (this happened twice). The infant continued on mixed feeding (mum continued to consume dairy products into her diet) with Frisolac AC and an OFC with the Standard Formula took place after 1 week. During the OFC he was given 90mL of the Standard Formula. Approximately 2.5 hours later he had profuse vomiting and discomfort. He fell asleep and when he woke up, he was fine suggesting the diagnosis of FPIES. He continued on Frisolac AC until the age of 15 months when he outgrew CMPA.																	
CSCy13	Male	Mixed fed	Normal	Normal	Father with Allergic Rhinitis and Mother with CMPA in infancy	2	1	Standard formula	Vomiting (FPIES)	Vomiting; Irritability/ Distress; Lethargy	4	1		NO			
The infant was EBF for the first 4 weeks of life. At the age of 1 month mother introduced a supplementary Standard Formula into his diet. After the first feedings infant developed increased irritability and discomfort. Given to his mother's hypersensitivity to cow's milk he was introduced to Frisolac AC. At the age of 4 months mother tried to reintroduce the Standard Formula into his diet but 2 hours later he had multiple vomiting, became lethargic and fell asleep. He woke up without any problem. Formula was switched again to Frisolac AC. Mother continued breastfeeding without excluding dairy products from her diet. At the age of 6 months, the infant had the same presentation after eating biological rice cream. Breastfeeding was discontinued at the age of 7 months and the infant is on Frisolac AC. He has not outgrown CMPA yet.																	
CSCy14	Male	Mixed fed	Normal	Caesarean	Father with hypersensitivity to fresh milk	1	1	Standard formula; Partially Hydrolysed formula	Vomiting (FPIES)	Vomiting; Irritability/ Distress	13	1	26.5	NO	18	17	23
The infant was mixed fed from the 2nd day of life with a Standard Formula. At the age of 3 months he developed increased irritability after feedings and then, the formula was switched to Frisolac AC and symptoms improved. Two weeks later Frisolac AC was switched to a partially hydrolysed infant formula. About 1.5 hour later he became distressed and had profuse vomiting (6 episodes). The infant formula was switched again to Frisolac AC. A month later (at age 4.5 months) mother tried another partially hydrolysed formula resulting again to intense irritability and vomiting 1.5 hour after feeding. Therefore, the infant continues on Frisolac AC until today. At the age of 12 months he had a similar episode after eating a cream with fresh cow's milk and fruits. At the age of 24 months he tolerated small amounts of baked milk, cheese and yoghurt. Mother is reluctant to give infant milk.																	
CSCy15	Male	Formula fed	Normal	Caesarean	Brother with CMPA	1	1	Partially Hydrolysed formula	Diarrhoea	Regurgitations; Vomiting; Diarrhoea; Irritability/ Distress; Atopic Dermatitis	1	4	18	NO		17	17

The infant was exclusively formula fed with Partially Hydrolysed formula from birth. At the 2nd day of life, he had intense irritability, multiple liquid stools, regurgitations, an episode of vomiting and mild facial rash. Infant formula was switched to Frisolac AC and symptoms resolved in 4 days. After a 2 weeks period of elimination diet, an OFC with the Partially Hydrolysed formula was performed and symptoms reappeared including atopic dermatitis thus, confirming CMPA diagnosis. Therefore, he continued on Frisolac AC without any problems. By the age of 12 months, the infant had tried cheese without any adverse event. At the age of 17 months OFC was performed with negative results confirming that he outgrew CMPA.																	
CoCy01	Male	Mixed fed	Normal	Caesarean	Mother and Sister with Allergic Rhinitis	2	0	Standard formula	Goat's type stools (Constipation)	Goat's type stools; Constipation; Irritability/ Discomfort;	2	7	3	YES	17	17	16.5
Infant was mixed fed with Standard Formula from the age of 1 week. At the age of 2 weeks, he developed constipation (goat's type stools), irritability and significant discomfort during defecation. Standard Formula was switched to Frisolac AC and his symptoms resolved and his motions became normal. Two weeks later an OFC took place and the Standard Formula was introduced back into his diet. Four days after the reintroduction his symptoms reappeared (4 days without motion, bloating, irritability and distress) and CMPA was confirmed. He continued on Frisolac AC and had occasionally mild gastrointestinal symptoms. However, at the age of 4 months, he became again very irritable and distressed with excessive crying during decefication. The formula was switched to AAF and his symptoms resolved completely in 2 weeks. He continued on AAF until the age of 17 months when he outgrew CMPA.																	
CoCy02	Female	Mixed fed	IVF	Caesarean	Sister with CMPA	1	1	Standard formula; Partially Hydrolysed eHF (whey based); Other type of special infant formula with hydrolysed protein	Constipation	Constipation; Irritability/ Distress	2	7	8.5	NO	12	12	11.5
The subjects FAECP022 and FACEP023 are twins. This infant was mixed fed for the first 3 days of life with a Standard Formula. The formula was switched to a Partially Hydrolysed formula because of her sister's symptoms of vomiting. At the age of 10 days formula was switched to another Standard Formula and she developed intense irritability, discomfort and constipation. The infant formula was switched again to 2 different special formulas with hydrolysed whey proteins but none of them improved her symptoms significantly. In addition to the mixed feeding, mother was giving to the infant probiotic supplements. Weight gain was within normal. At the age of 3.5 months formula was switched to Frisolac AC and symptoms resolved after 1 week while she was breastfeeding without exclusion of dairy products in mother's diet. From the age of 4 months, breastfeeding was discontinued and the infant was exclusively formula fed with Frisolac AC until the age of 12 months when she outgrew CMPA.																	

CoCy03	Female	Mixed fed	IVF	Caesarean	Sister with CMPA	1	1	Standard formula; Partially hydrolysed Formula; Other type of infant formula with hydrolysed protein	Constipation	Regurgitations; Constipation; Irritability/ Distress	1	7	8.5	NO	12	12	12
The subjects FAECP022 and FACEP023 are twins. This infant was mixed fed for the first 3 days of life with a Standard Formula. In the first week of life she developed vomiting with every feeding and the infant formula was switched to a Partially Hydrolysed formula. At the age of 10 days formula was switched to another Standard Formula and she developed intense irritability, discomfort and constipation. The infant formula was switched again to 2 different special formulas with hydrolysed whey proteins but none of them improved infant's symptoms significantly. In addition to the mixed feeding, mother was giving the infant probiotics supplements. Weight gain was within normal. At the age of 3.5 months formula was switched to Frisolac AC and symptoms resolved after 1 week. The infant was mixed fed with Frisolac AC up to 7 months of age while her mother was consuming dairy products. From the age of 7 months, the infant was exclusively formula fed with Frisolac AC until the age of 12 months when she outgrew CMPA.																	
CoCy04	Female	Mixed fed	Normal	Caesarean	Mother with hypersensitivity to cow's milk in childhood	1	1	Partially Hydrolysed formula	Constipation	Regurgitations; Constipation; Goat's type stools; Irritability/ Distress; Poor weight gain	3	10	8	NO	12	12	11
The infant was mixed fed from birth, with a Partially Hydrolysed formula because the mother had a history of cow's milk hypersensitivity in early childhood. At the age of 3 weeks the infant developed regurgitations with every feeding. Breastfeeding was discontinued at age 5 weeks and as regurgitations and discomfort were increasing, mother was advised to alternately feed her with a Standard Formula and Frisolac AC. Regurgitations worsen and the infant also developed irritability, discomfort and constipation. Then, mother was advised to switch Standard Formula with a Special Infant Formula for regurgitations (and continue alternate feeding). Symptoms did not improve and at the age of 3.5 months she was seen in the allergy clinic and was advised to continue only with Frisolac AC. Symptoms resolved completely within 10 days. She was on Frisolac AC until the age of 12 months when she outgrew CMPA.																	
CoCy05	Male	Mixed fed	Normal	Caesarean	None	0	0	Standard formula; Anti-Reflux; Lactose free formula	Constipation	Constipation; Diarrhoea; Irritability/ Distress	1	N/A	0.5	YES		14	14

The infant was mixed fed with a Standard Formula from the first days of life. During the 1st week of life, he developed irritability, distress and difficulty in decefication. He could have a motion only after the use of a glycerine suppositor. At the age of 2 weeks he also developed regurgitation and had a possible episode of suffocation. The infant formula was switched to 2 different Special Formulas for regurgitations without any improvement. The following week the infant was exclusively breastfed and then mother tried to introduce a Lactose-free infant formula into his diet. His symptoms did not resolve and the infant formula was switched to Frisolac AC. However, the infant developed diarrhoea, irritability and distress and a few days later infant formula was changed to an AAF. His symptoms resolved in 3 days. The infant was mixed fed until 6 months of life (breast milk and AAF) and then he was exclusively formula fed until the age of 14 months when he outgrew CMPA.

Footnote:

CSCy01-11: Patients with FPIAP

CSCy12-14: Patients with FPIES

CSCy15: Patient with CMPA-related Diarrhoea

CoCy01-05: Patients with CMPA-related Constipation

CSCy04, CoCy01 and CoCy05: Patients required AAF
